# Multiscale structural complexity as a quantitative measure of visual complexity

**DOI:** 10.1177/03010066251384492

**Published:** 2025-11-20

**Authors:** Anna Kravchenko, Andrey A Bagrov, Mikhail I Katsnelson, Veronica Dudarev

**Affiliations:** 1 6029 Radboud University, Nijmegen, the Netherlands; 2 8166 University of British Columbia, Vancouver, Canada

**Keywords:** complexity, visual complexity, vision, modeling

## Abstract

While intuitive for humans, the concept of visual complexity is hard to define and quantify formally. We suggest adopting the multiscale structural complexity (MSSC) measure, an approach that defines structural complexity of an object as the amount of dissimilarities between distinct scales in its hierarchical organization. In this work, we apply MSSC to the case of visual stimuli, using an open dataset of images with subjective complexity scores obtained from human participants (SAVOIAS). We demonstrate that MSSC correlates with subjective complexity on par with other computational complexity measures, while being more intuitive by definition, consistent across categories of images, and easier to compute. We discuss objective and subjective elements inherently present in human perception of complexity and the domains where the two are more likely to diverge. We show how the multiscale nature of MSSC allows further investigation of complexity as it is perceived by humans.

## Introduction

In studying information processing in human perception, attention, and thinking, we are often faced with the issue of characterizing the information to be processed in objective terms. One of the earliest steps in perception research—what we now know as psychophysics—aimed at discovering the laws that connect perceived properties of stimuli to their physical properties, such as brightness of a dot, loudness or pitch of a sound. The relationships between subjectively perceived and objective complexity could be studied as well, and could inform studies on downstream effects of complexity, for example, on esthetics, attention, and motivation (Berlyne, 1960; de Winter et al., 2023; Kondyli et al., 2023).

For example, visual complexity is an important factor in determining esthetic preference for artistic works (Forsythe et al., 2011; Jacobsen & Höfel, 2002). Early works suggested a linear relation between visual complexity and esthetic beauty (Eysenck, 1941; Reinecke et al., 2013), where perceived beauty is proportional to esthetic order but decreases with increased complexity (Birkhoff, 1933). However, later studies revealed that the relationship is more of an optimum type: most appealing images tend to have intermediate levels of visual complexity (Berlyne, 1971; Vitz, 1966).

Curiosity is another field where the complexity of information is an important factor, along with novelty and learning difficulty (Berlyne, 1960; Kidd & Hayden, 2015). While curiosity was shown to improve learning speed and outcomes in AI systems (Gottlieb & Oudeyer, 2018; Oudeyer, 2018; Twomey & Westermann, 2017), AI models implementing curiosity measures relying on complexity and novelty seem to be disrupted by noise (Burda et al., 2018; Oudeyer, 2018), and more recent research sees the preference for intermediate complexity as coincidental with optimizing learning progress (Poli et al., 2024).

Characterizing complexity objectively, however, has been a persistent issue, not only in psychology. First attempts to quantify complexity arose in coding theory and data compression, focusing on statistical properties of data and the amount of information needed to reliably transmit it in a message. Shannon (1948), quantified information through entropy, the amount of uncertainty present in a message, while Kolmogorov defined complexity as the length of the description needed to reproduce the stimulus (Kolmogorov, 1968; Sun & Firestone, 2022).

**Figure 1.**
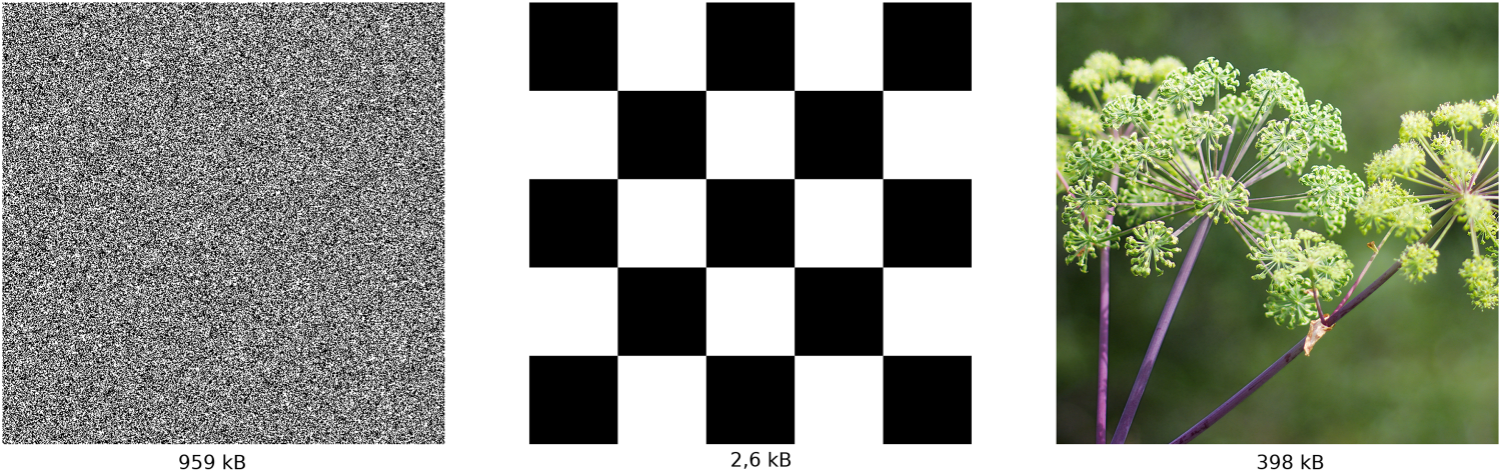
Descriptive measures of complexity tend to prioritize pure randomness over structure, ranking noise as more complex than meaningful images.

These measures are usually referred to as measures of informational complexity and they focus on the amount of randomness present in data rather than structural nontriviality. There are multiple ways to implement these concepts. For example, file size (Machado et al. (2015) can be viewed as an upper bound on Kolmogorov’s complexity of its content. To an extent, these measures do correlate with ratings of complexity provided by human observers (de Winter et al., 2023; Machado et al., 2015; Saraee et al., 2020), however, they also assign high complexity to random stimuli (Figure 1, left), which are not perceived as complex. Meanwhile, things that we humans perceive as truly complex tend to have a balance between order and randomness (Figure 1, right), that is, they are structurally nontrivial.

In psychological literature, several qualitative analyses of complexity describe it as a function of three factors: the number of elements (which is still consistent with Kolmogorov and Shannon), the dissimilarity between the elements, and their organization within the stimulus (Berlyne, 1960; Donderi, 2006; Oliva et al., 2004). For abstract black-and-white patterns, Chipman (Chipman, 1977) introduced the distinction between “quantitative” and “structural” variables underlying perceived complexity, the latter being a combination of a number of measures of symmetry, repetitions, and so on. For more naturalistic and complex images like photographs and art, dissimilarity and organization proved to be even harder to quantify computationally. Edge density and visual clutter (Oliva & Mack, 2004; Rosenholtz et al., 2007) are some examples of the attempts to do so. However, defining dissimilarity and especially organization in a way that is generalizable across different types of stimuli is challenging (Donderi, 2006). One of the most successful attempts to quantify this aspect of complexity is a measure of the ‘‘number of regions” (Comaniciu & Meer, 2002). This computational measure correlates quite well with subjective complexity (Saraee et al., 2020), especially in combination with other measures (Nuthmann & Einhäuser, 2015). Its downside is that it requires some user input in setting the parameters, without clear guidelines on how to do that.

So far we have reviewed the attempts to quantify complexity as a single variable. Oliva et al. (2004) suggested that complexity is not a uni-dimensional metric. Yet to the best of our knowledge no computational model of dimensional measure of complexity has been proposed for naturalistic images (for abstract patterns, see Chipman, 1977). In some way, multiscale structural complexity can be considered as a first step in this direction, as we show below.

Another persistent issue in quantifying complexity is the relationship between computational complexity and complexity as it is perceived by humans. While developers of computational measures of complexity aim at a perfect correlation with perceived complexity, one could theorize that perfect agreement is not—and should not be—possible. This is because perceived complexity, as any perceived quality, arises from the interaction between the objective properties of the stimulus and characteristics of the observer, for example, observer’s prior experience, familiarity with the stimulus, and the specific task or purpose guiding perception. The best example of this is characters in a language one can versus cannot read. A mathematical formula, while being a fairly simple visual pattern, might convey incredibly detailed information. Recognizability of the pattern, being a property of the perceiver, not the perceived, is therefore impossible to predict algorithmically (Spehar et al., 2015). Madan et al. (2018) showed that human ratings of complexity are inherently influenced by affective value the observer ascribes to the stimulus. That is, what we see as more emotional is rated higher on complexity. Together, these examples suggest that perceived complexity does not reflect just the number, dissimilarity, and organization of the elements, but includes additional factors. These additional factors could be the reason why most of the existing measures of computational complexity show different magnitude of correlation with perceived complexity for photographs as compared to paintings, the latter presumably having more prominent emotional and cultural aspects (Saraee et al., 2020). Some researchers (Oliva et al., 2004; Saraee et al., 2020) go as far as to conclude that different computational measures of complexity are more suitable for different domains (stimulus types).

The notion of complexity also happens to be important in physics where it is used to describe emergent phenomena in systems of multiple components (Bagrov et al., 2020). In this work, we use a measure called multiscale structural complexity (MSSC), and apply it to the case of visual stimuli. We compare it to other computational measures of complexity and perceived complexity, and demonstrate how its multiscale nature can help investigate human perception of image complexity.

## Multiscale Structural Complexity

One of the hallmarks of the majority of complex systems observed in the world—from biological structures to pieces of art—is the co-existence of a number of well-defined characteristic scales. In other words, most complex systems have hierarchical organization (Broido & Clauset, 2019). For example, any living organism is structured in a multilevel way, with levels of organs, tissues, cells, sub-cell organelles, and complex molecules reaction networks being fundamentally different from each other. It was suggested that competing interactions between these levels is what gives rise to physical and biological complexity (Wolf et al., 2018).

A similar idea has been suggested for image perception by the scale-space theory (Lindeberg, 2008). Namely, this framework emphasizes that real-world objects may be perceived differently at different scales of observation (i.e., from close up vs. from afar). It was further suggested that some of the scales may be more informative for a particular task and less informative for another one, hence an efficient computer vision system would benefit from having access to representations at the different scales (Lindeberg, 2008). We suggest that the number of potentially meaningful nonredundant scales present in the image and their distribution are important components of complexity.

The idea of complexity as self-dissimilarity at different spatial or temporal levels has been embraced by Wolpert and Macready (1997) and Wolpert and Macready (2007). Following this line of thinking, the concept of MSSC has been introduced (Bagrov et al., 2020). MSSC originated in physics and was first used to describe phase transitions in classical and quantum systems composed of many components. At the same time, since it was inspired by the intuitive human perception of complexity, applying it in the realm of visual perception is a natural endeavor, which we pursue in this article.

**Figure 2.**
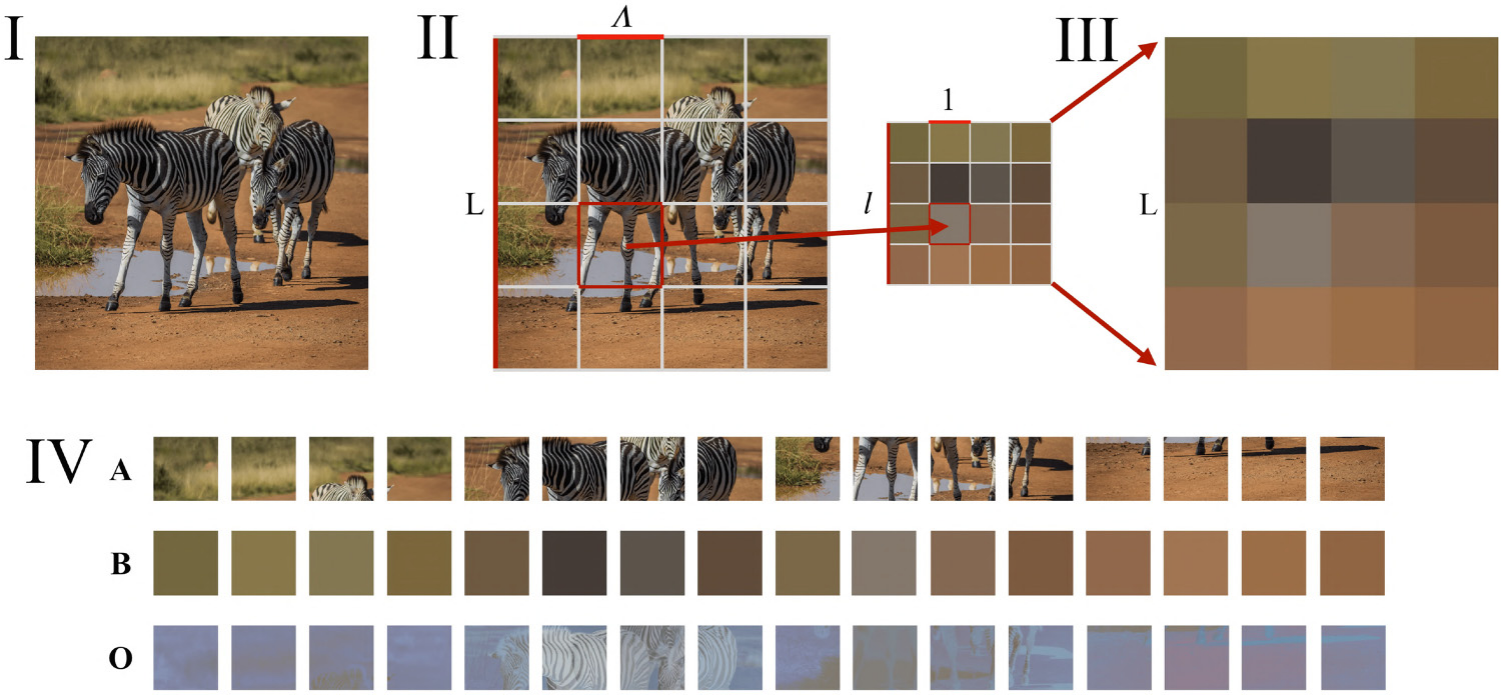
From Bagrov et al. (2020): Schematic representation of the idea behind the coarse-graining method. The difference between versions of the image at each step of coarse graining determines partial complexity contributed by the spatial scale removed at that step.

MSSC quantifies the amount of distinct scales present in a visual pattern using the idea of coarse graining, or renormalization group (RG), borrowed from physics. The concept of RG has been introduced in physics to formally quantify how properties of a system depend on the scale at which it is probed (Gell-Mann and Low, 1954). An accessible and detailed introduction to the RG is given by Ma (1973), and an example of how it can be applied to image processing can be found in Gidas (1989). The formal mathematical definition of MSSC is provided in the Appendix. Here we briefly explain how it works on a more conceptual level. Step by step, information is erased from the pattern—first from the most detailed microscopic scale, and then from larger and larger scales, as shown in Figure 2. This generates a stack of patterns $P_{i}$ derived from the original image (denoted as $P_{0}$ ). Now, assume that we compare two subsequent patterns in this stack, $P_{k}$ and $P_{k+1}$ . If the difference between them is substantial, it implies that considerable amount of information has been lost at the coarse-graining step k+1 . This, in accordance with the idea of multiscale dissimilarity, would produce a large value of *partial complexity* $C_{k}$ to scale k . On the other hand, if $P_{k}$ and $P_{k+1}$ are nearly identical, scale k does not bear any unique features, and complexity value associated with it is low. Cumulative sum of partial complexities over relevant scales $C=\sum_{k}C_{k}$ is called multiscale structural complexity.

MSSC should be regarded as an umbrella concept rather than a singular definition, as it can be implemented in multiple ways. First of all, the coarse-graining procedure can be performed in a variety of ways. At this stage of MSSC development, the choice should be guided by theoretical considerations of which approach best suits the problem. For example, in the original article, it has been shown that even something as simple as averaging over segments of a picture as shown in Figure 2 provides sufficient results when addressing the problem of phase transitions in complex physical systems. Topology simplification (Batavia et al., 2021) could also serve as a promising alternative. In the current work, focusing on visual complexity, we chose an approach that is biologically plausible for human visual perception (see details in the next section).

The second aspect is that not all scales formally present in the pattern are relevant and have to be taken into account. According to Bagrov et al. (2020), it was shown that better results in identifying phase and structural transitions can be achieved if one neglects the smallest scales, where the very notion of structure and correlation length is not established yet, and the largest scales that exceed the maximal characteristic length of pattern features. In this article, we will study how partial complexities of different scales of a visual stimulus correlate with the human ranking, and show that the best practice is indeed to account for a particular range of scales when computing C .

## Implementation

It is possible to implement coarse graining in a variety of ways, which allows us to aim for the more biologically plausible approach. Evidence suggests processing on early layers of the visual cortex can be approximated by Fourier transform (Campbell & Robson, 1968; Kesserwani, 2020; Kulikowski & Bishop, 1981; Ochs, 1979; Olshausen, 2003; Stevens, 2004). Fourier transform analyses a signal into frequencies that compile it. An image would be Fourier-transformed into a sum of spatial frequencies, where high-frequency components correspond to fine details, textures and edges, and low-frequency components correspond to larger shapes and smooth, gradual variations in intensity. Fourier transform is often used to characterize and manipulate visual stimuli in cognitive science (Marr, 2010). Here we use it to perform coarse graining.

Namely, we decompose the image into spatial frequencies by applying discrete Fourier transform (Equation (1)). Then, step by step, we remove from the sum of the spatial frequencies the highest band (this is called low-pass filtering) and reconstruct the image from what remains (Equation (2)):

(1) $$F_{k_{x},k_{y}}=\sum_{n_{x},n_{y}=0}^{N-1}f_{n_{x},n_{y}}\cdot e^{-i2\pi \frac{k_{x}n_{x}+k_{y}n_{y}}{N}},$$

(2) $${\tilde{\;f}}_{n_{x},n_{y}}=\frac{1}{N^{2}}\sum_{k_{x},k_{y}=0}^{N-K-1}F_{k_{x},k_{y}}\cdot e^{i2\pi \frac{k_{x}n_{x}+k_{y}n_{y}}{N}},$$

where $f_{n_{x},n_{y}}$ is the intensity of pixels in the original gray-scale two-dimensional image (with $n_{x},n_{y}$ pixel co-ordinates), and ${\tilde{\;f}}_{n_{x},n_{y}}$ is its coarse-grained version obtained by removing K highest frequencies.

**Figure 3.**
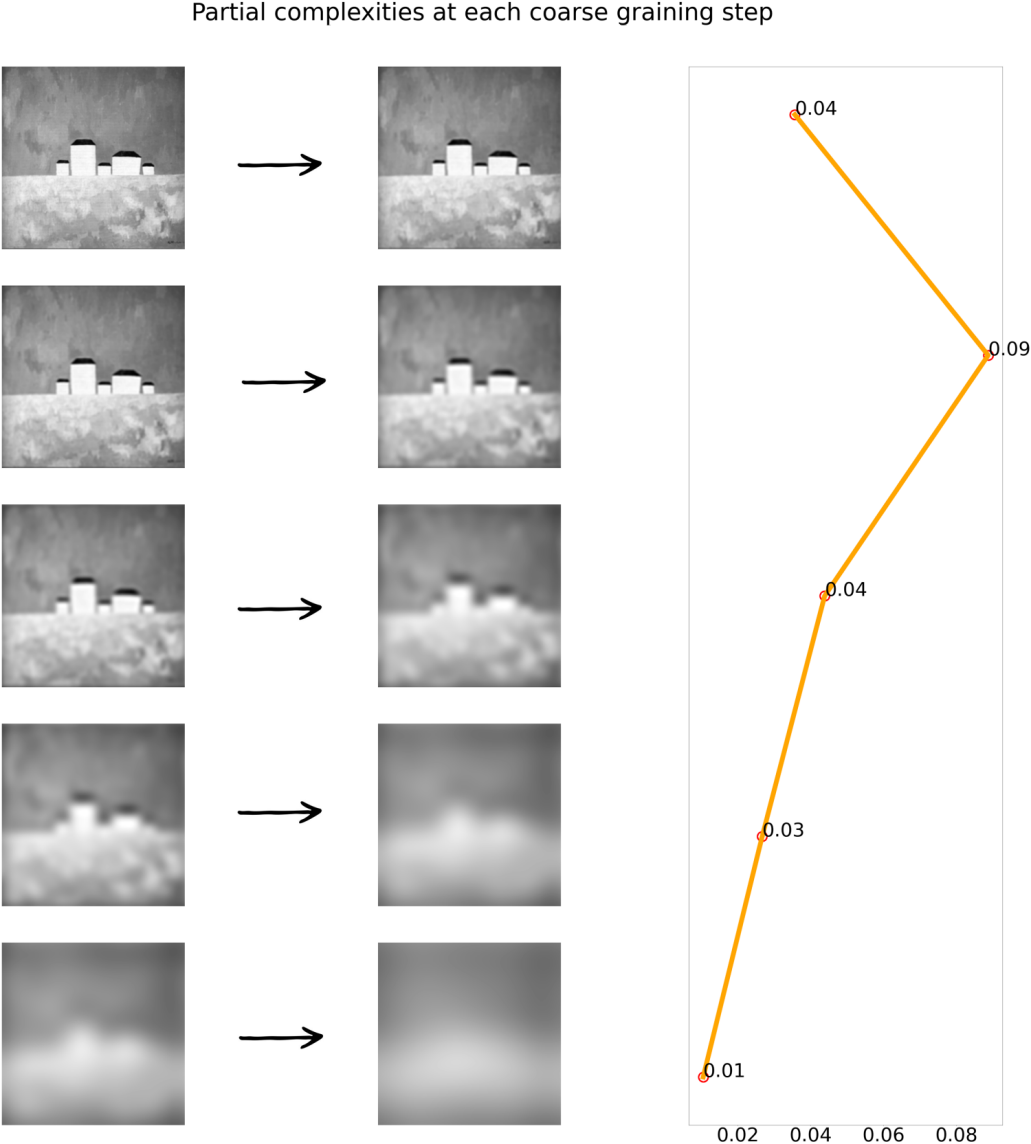
Left Panel: Visualization of steps of the coarse-graining procedure performed with fourier transform. Right Panel: Partial complexities $C_{k}$ contributing to overall complexity. Partial complexity can be regarded as the amount of information that gets lost at a coarse-graining step. Total complexity of the image is the sum of partial complexities. for illustrational purposes only five coarse-graining steps were used to highlight the changes at each iteration of coarse graining. low-pass filter radii for calculating the cutoff frequency at each step were spaced evenly on a log scale from 0 to 512 px (length of the image side).

In the current implementation of the algorithm, each image was reshaped to 512 × 512 px and we performed 10 coarse-graining steps. Low-pass filter radii used to calculate the cutoff spatial frequency at each step were spaced evenly on a log scale from 0 to 512 px. A good informal educational explanation of low-pass filtering with Fourier transform can be found at Tutorials (2019). An example of the process can be seen in Figure 3.

The code for the current implementation can be found at: https://github.com/ankravchenko/mssc/tree/master.

## The Present Study

We used a published set of images with computational and subjective complexity measures—Scenes, Advertisement, Visualization and infographics, Objects, Interior design, Art, and Suprematism (SAVOIAS) (Saraee et al., 2020) to estimate the correlation between their human ranked complexities and MSSC values. We selected this dataset because it is an open source one that provides access to subjective complexity value for each image. In addition, Saraee et al. (2020) supplement subjective ratings with computational measures of complexity, which allows us to compare MSSC to those as well.

### Dataset

**Figure 4.**
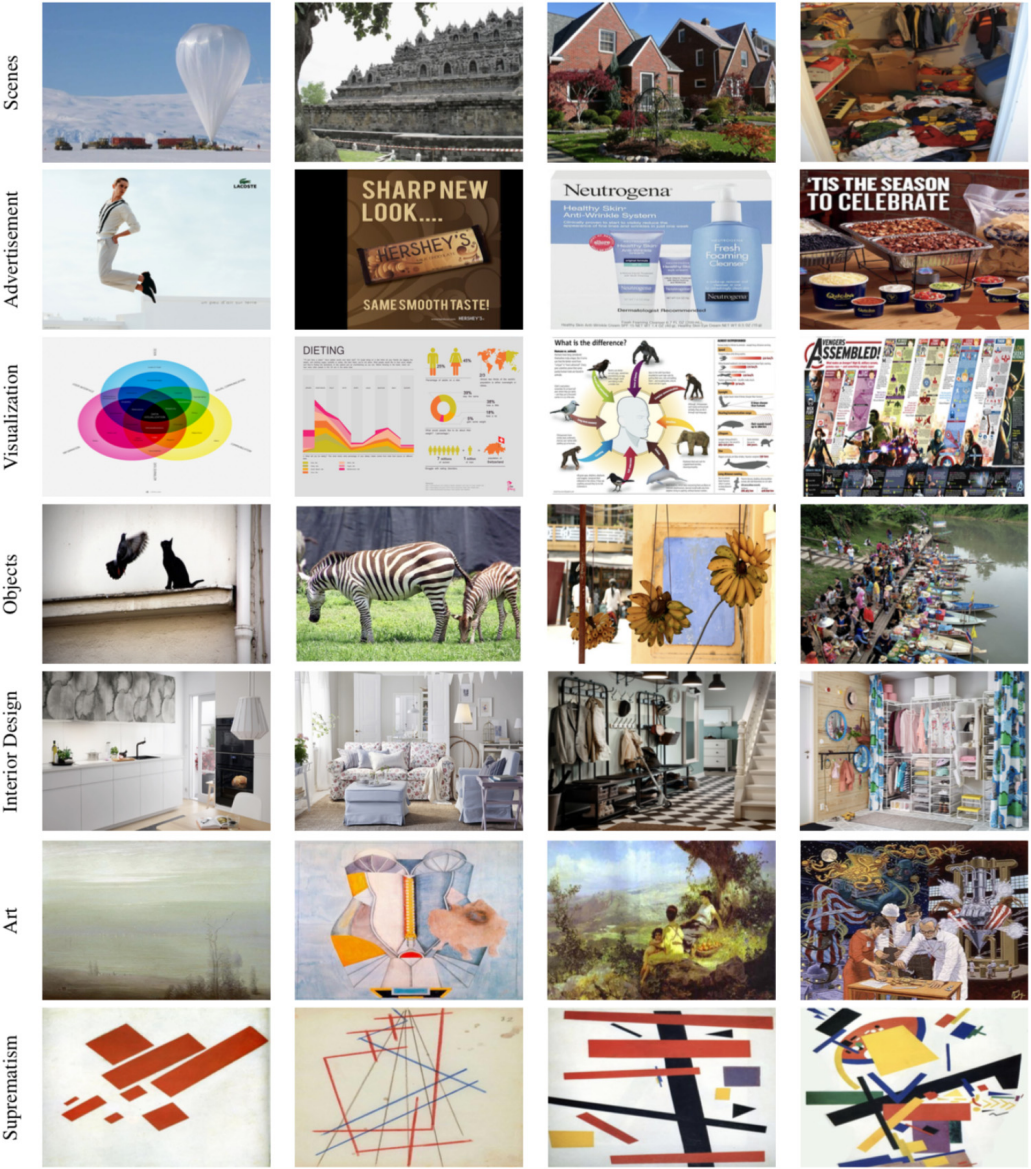
Example images from SAVOIAS dataset for the categories of: Scenes, Advertisement, Visualization and Infographics, Objects, Interior Design, Art, and Suprematism (Saraee et al., 2018).

SAVOIAS (Saraee et al., 2020) is a set of 1,420 images grouped into seven categories (Figure 4): Scenes (photographs of natural scenes), Advertisements, Visualization and Infographics, Objects (photographs of objects in natural context), Interior design (photographs of interior design displays from Ikea cataglogue), Art, and Suprematism (the distinction between Art and Suprematism was introduced by Saraee et al. (2020), we keep this distinction for consistency).

Subjective complexity for these images was obtained from 1,687 participants in the following way: participants were presented with pairs of images and were asked to select the more complex one. Each participant was presented with a subset of images, all coming from the same category. In total, 37,000 of image pairs were considered by participants, and the relative scores were then converted to a continuous measure of visual complexity using the (Bradley & Terry, 1952) method and matrix completion by Candes and Recht (2012). It was shown that the correlation between resulting measures based on complete all-to-all pairwise comparisons within a category and the ones based on partial comparisons becomes nearly perfect ( r>.95 ) for as few as ∼2,000 compared pairs within a specific category. For full details about the image selection, the subjective and objective measures of complexity, as well as for a detailed discussion of the statistical reliability of partial pairwise comparison, see Saraee et al. (2020).

### Methods

MSSC was computed for each image in the way described above (see the Implementation section, the code is available at https://github.com/ankravchenko/mssc/tree/master). We processed each color channel separately, calculating its intensity before estimating complexity of this channel and then summed the resulting complexities multiplied by intensity for all channels.

As we explained above, partial complexities at each step of coarse graining may have varying impact on overall complexity. They are also not equally meaningful. Elements present only at the smallest scales are likely image artifacts or details insignificant to the human eye, while later steps of coarse graining, at which objects start to disappear, happen on the scales comparable to the scale of image itself. For the smallest and largest scales, it is then likely that computational complexity (dissimilarity) will be large, yet the content of the image—hence perceived complexity—will not change. To account for that in our analysis we started with investigating partial complexity at each step of coarse graining and its correlation with perceived complexity. We then selected cutoff points for the smallest and the largest frequencies, with the restriction that the same threshold is used across all image categories, and computed MSSC as a sum of partial complexities for the remaining middle scales. We then computed Pearson correlation between MSSC and perceived complexity provided by Saraee et al. (2020).

## Results

**Figure 5.**
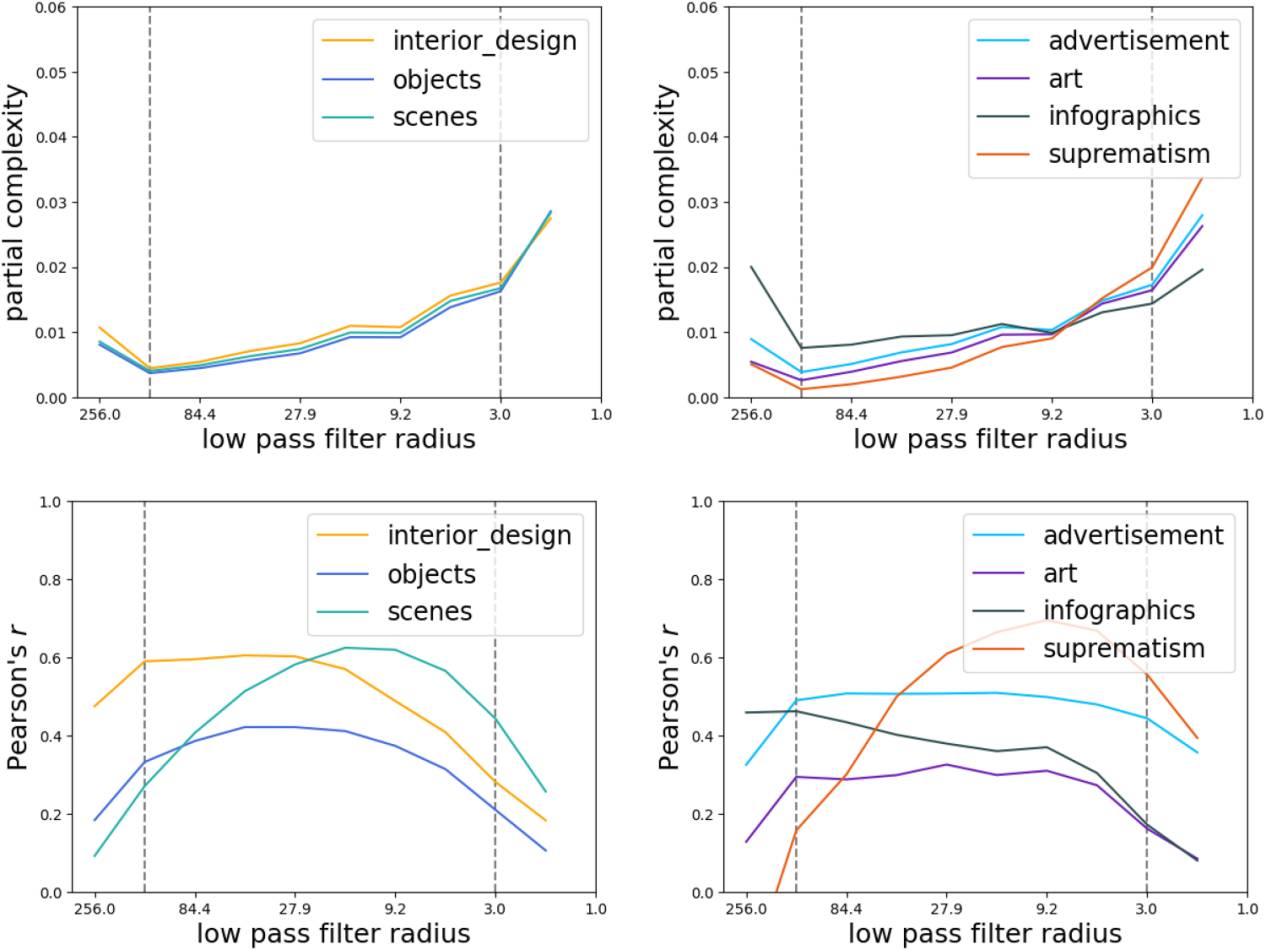
Partial complexity at each step of coarse graining (top row) and its correlation with subjective complexity (bottom row) for the different categories (shown in color). The vertical lines show the cutoff points: only the steps between the two lines were included in multiscale structural complexity (MSSC). Low-pass filter radii for coarse-graining steps for images of 512 × 512 px were spaced evenly on a log scale.

As anticipated, the smallest and the largest frequencies produced relatively high partial complexity scores (Figure 5, top row) that had relatively low correlations with perceived complexity (Figure 5, bottom row), across all image categories. We therefore computed MSSC including only the middle scales (between the two vertical lines in Figure 5).

**Figure 6.**
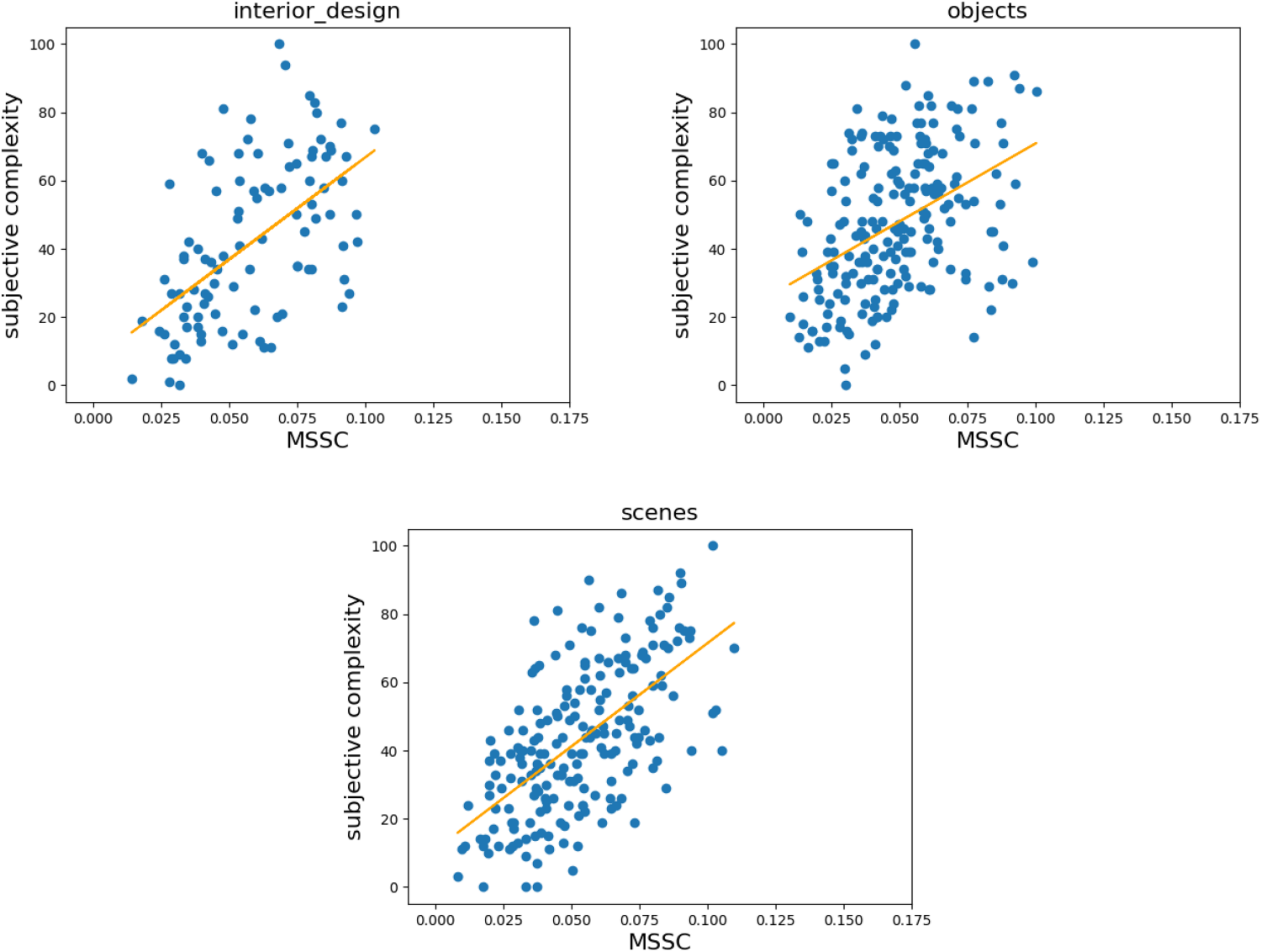
Linear regression analysis (Freedman, 2005) of correlations between subjective complexity (vertical axis) and multiscale structural complexity (MSSC) (horizontal axis) of the images. Each point on the scatter plot represents an image from the chosen category within the dataset, and the orange line shows the linear fit of the data. Here we focus on scenes, objects, and interior design, which we heuristically call natural scenes.

**Figure 7.**
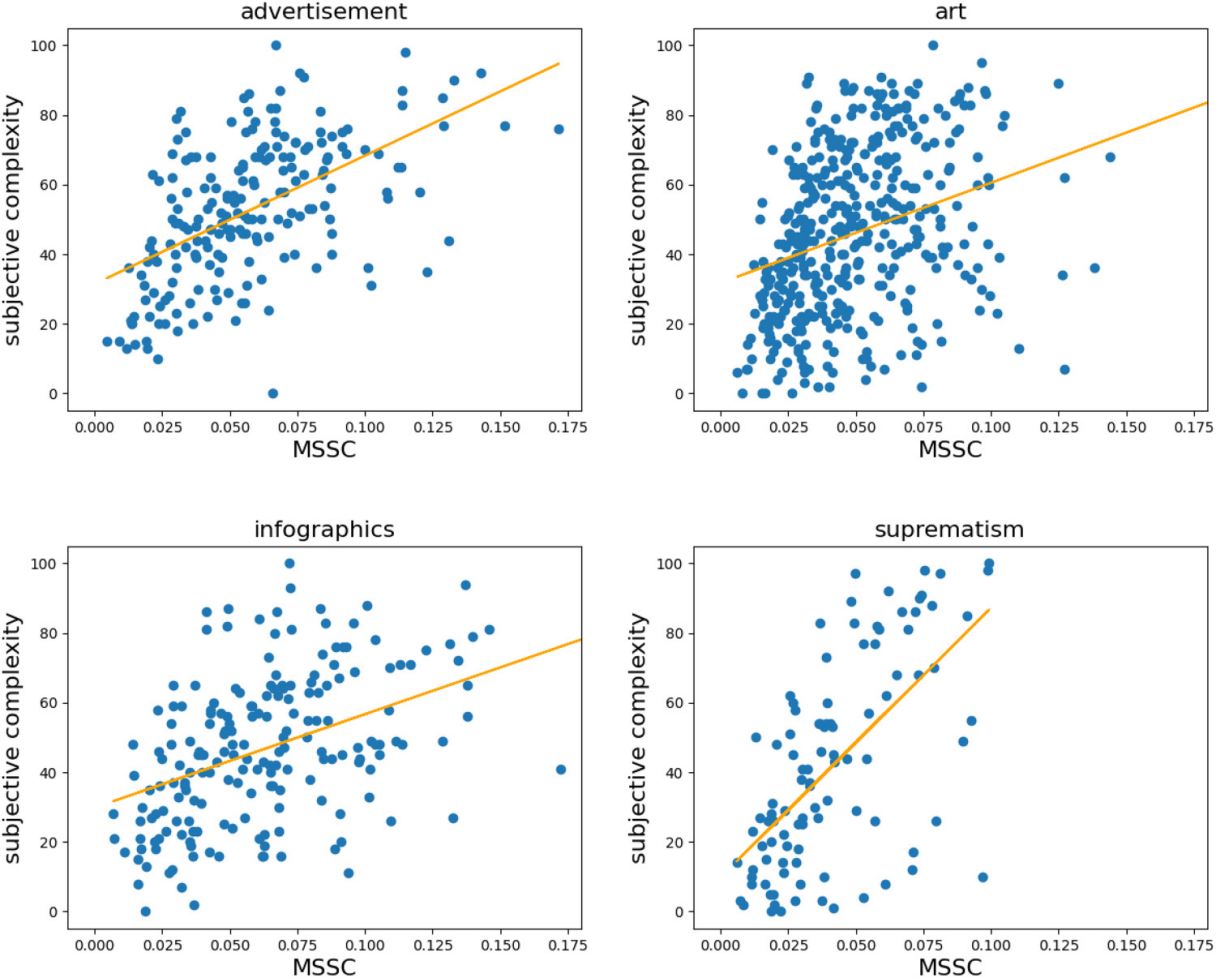
Subjective complexity (*y* axis) predicted by multiscale structural complexity (MSSC) (*x* axis) for advertisements, art, infographics, and suprematism, which we heuristically call man-made images. The scatterplot for art does not show two images for which MSSC was >2, although they were included in all the reported analyses. For more information on these two images, see the Discussion section.

Figures 6 and 7 show the scatterplots for the correlations between MSSC and subjective complexity for each category, which we further divided into two clusters: “Natural scenes” refer to images obtained by means of photography, while “Man-made images” refer to images painted or digitally produced by humans. Although this division is purely heuristic, the figures show that man-made images produce noisier correlations between MSSC and subjective complexity. This is especially evident for the “art” category, where MSSC assigns a high value to what humans perceive as not very complex. We will return to this point in the Discussion section.

**Figure 8.**
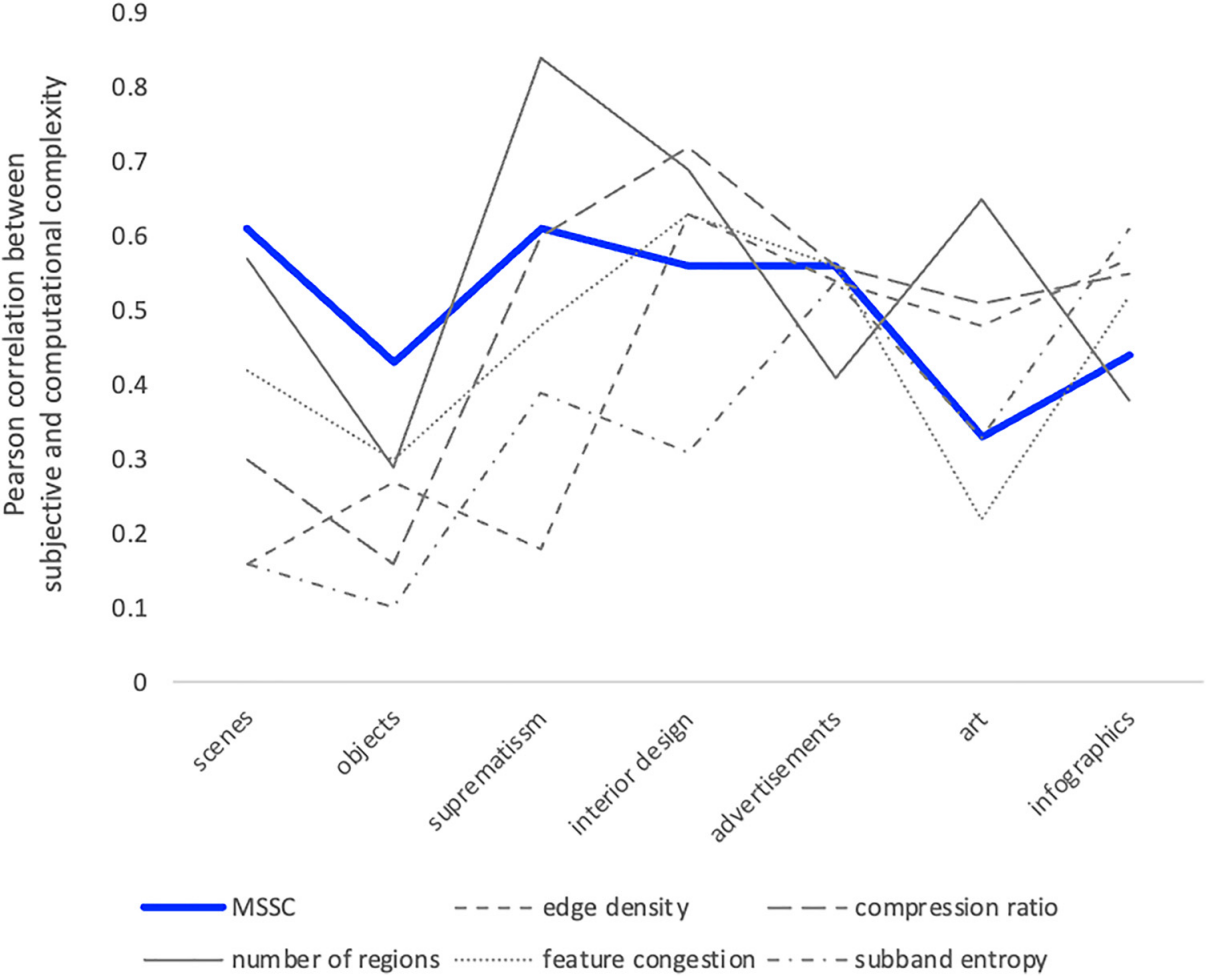
Pearson correlations between subjective complexity and computational measures of complexity, cisualized. We can see that multiscale structural complexity (MSSC) (blue line) behaves more consistently across different domains.

Table 1 shows the Pearson correlation between MSSC and subjective complexity (the first column). It also shows correlations from Saraee et al. (2020), between subjective complexity and other computational measures of complexity. The same data is visualized in Figure 8.

**Table 1.** Pearson correlations between subjective complexity and computational measures of complexity: multiscale structural complexity (MSSC) as computed in the present work, edge density and other measures taken from Saraee et al. (2020).

	MSSC	Edge density	Compression ratio	Number of regions	Feature congestion	Subband entropy
Scenes	**0.61**	0.16	0.3	0.57	0.42	0.16
Objects	**0.43**	0.27	0.16	0.29	0.3	0.1
Suprematism	0.61	0.18	0.6	**0.84**	0.48	0.39
Interior design	0.56	0.63	**0.72**	0.69	0.63	0.31
Advertisements	**0.56**	0.54	**0.56**	0.41	**0.56**	0.54
Art	0.33	0.48	0.51	**0.65**	0.22	0.33
Infographics	0.44	0.57	0.55	0.38	0.52	**0.61**

The highest correlation (numerically) is highlighted in bold font.

It is noteworthy that, descriptively speaking, MSSC agreement with subjective complexity varied the least between the different categories (Figure 8). For example, while the number of regions performed exceptionally well for art and suprematism categories, its correlation with subjective complexity was the lowest of all computational measures for advertisements, infographics, and it was quite poor for objects (only r=.29 ). That is, the agreement between the number of regions and subjective complexity varied widely across the categories, and the same was true for the other computational measures. MSSC had the lowest spread, showing higher consistency across categories than other computational measures of complexity. It also outperformed all the other computational measures for the scenes and objects categories (Table 1 and Figure 8. For the scenes, Pearson’s correlation between MSSC and subjective complexity was 0.61 , which was close, but slightly higher than the maximal 0.57 achieved by the other computational measures (number of regions). Objects were the most challenging category for all computational measures, with the maximal correlation for previous measures being just 0.3 , while MSSC reached 0.43 .

## Discussion

We have found significant correlations between MSSC and subjective complexity. MSSC performs on par with other computational methods in most cases, surpassing them on natural images and providing more consistent results across categories.

Having said that, it is evident that MSSC was not equally correlated with subjective complexity across categories. This, however, is true for all the other computational complexity measures. Saraee et al. (2020) suggested that no computational measures of complexity are generalizable across different categories of images, and proposed creating specialized metrics for every domain. This echoes the suggestion that complexity may not be a uni-dimensional measure (Oliva et al., 2004). The approach we introduce here and its implementation in MSSC opens the venue for formulating testable hypotheses about special concepts of complexity for each case.

**Figure 9.**
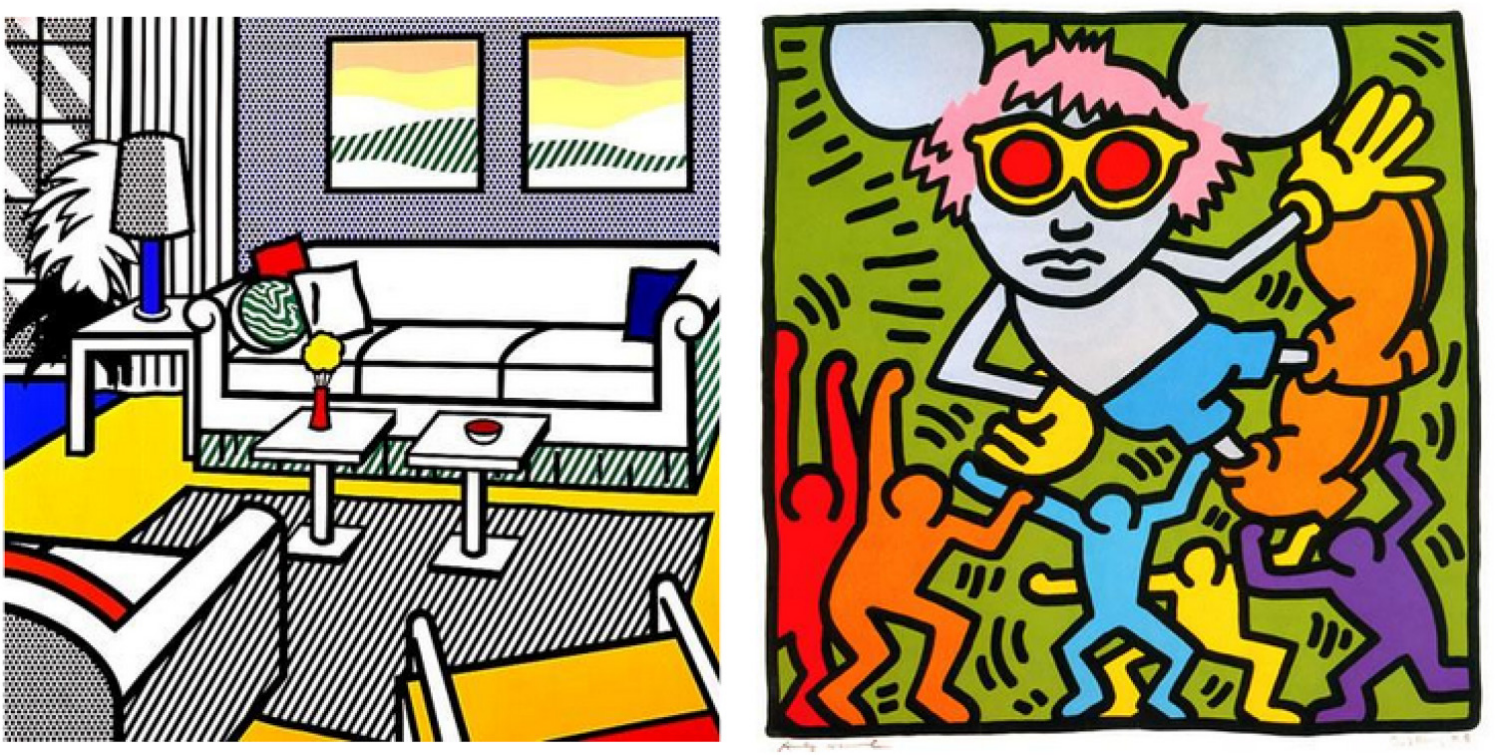
Examples of the images from the art category with unreasonably high multiscale structural complexity (MSSC) values (0.23 on the left and 0.2 on the right, top percentile). Subjective complexity was estimated as 48 and 57 for the left and right images.

For example, let us consider the impact of the partial complexity at different levels (steps). We anticipated that the lowest and highest spatial frequencies would disproportionately increase computed complexity compared to perceived complexity, and this is indeed what we found. Yet even after excluding extreme spatial frequencies from the MSSC, we see that for some images, especially in the ‘‘art” and ‘‘infographics” categories, MSSC was much higher than perceived complexity (see Figure 7, ‘‘art” and ‘‘infographics”). Figure 9 shows two examples of such images. In this case, MSSC was evidently overwhelmed by the small details, yet human participants largely ignored them in their complexity judgments. On the other hand, for art on average, the impact of smaller and larger spatial frequencies on perceived complexity was more uniform than, for example, for natural scenes. It is thus possible that human viewers weigh the amount of attention they pay to smaller details by the intentionality they assume behind these details, paying more attention to them when these details are expected to be informative. Notice that for three out of four man-made categories the relationship between partial complexity and perceived complexity was flatter than for natural images. Suprematism, however, showed a relationship similar to natural scenes, which could probably be taken as a sign of success of the artists’ mission to express “the supremacy of pure feeling or perception in the pictorial arts” (Malevich, Suprematist Manifesto, 1927, by Danchev, 2011).

**Figure 10.**
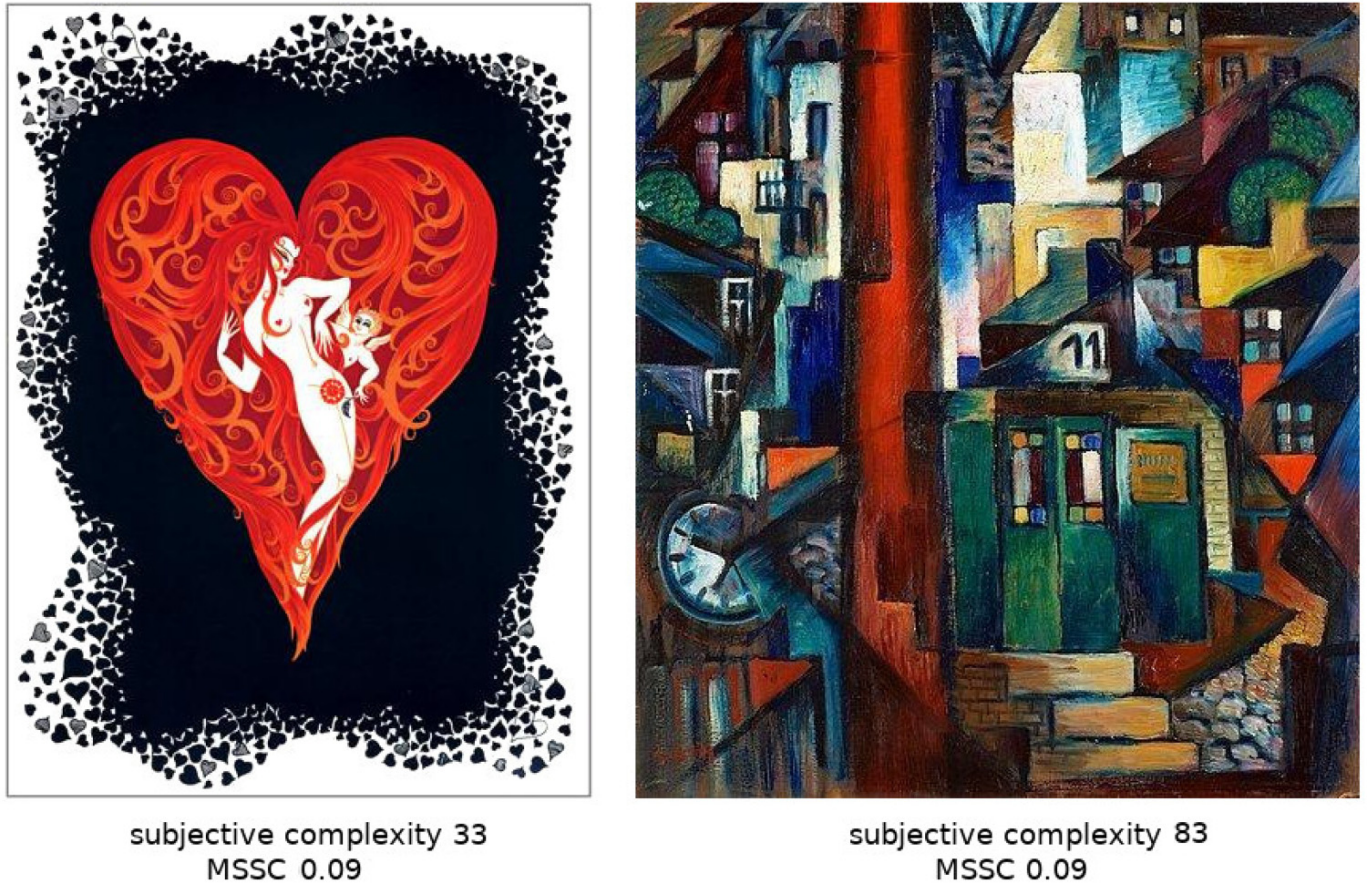
Example of images with the same multiscale structural complexity (MSSC) score that were ranked vastly differently by human participants. We attribute the difference to subjective estimates of artistic value and ‘‘message” conveyed by the picture.

Having said that, the fit between perceived and partial complexity was numerically lower for man-made images across all the frequencies, including the mid-scales. It is plausible that this could be attributed to the lack of general cultural knowledge humans use in viewing these types of images. In semiotic terms, when arbitrary, symbolic signs are present, their purely visual complexity will not reflect the complexity of the derived interpretation. An example of this is shown in Figure 10. For such images, complexity arises in the space of interpretations, not visual composition. It is possible that subjective complexity for images that rely on cultural knowledge would be more variable between the raters, which would naturally diminish the correlations with objective complexity.

A prominent limitation of the present study is how subjective complexity was measured by Saraee et al. (2020). Using aggregated two-alternative forced-choice responses regarding image complexity limited the extent to which we could test variability between raters in different image categories. Likewise, it was not in the scope of the present work to test whether asking participants to focus on perceptual—as opposed to conceptual—complexity would make a difference (Madan et al., 2018). We would expect that emphasis on perceptual complexity in the instructions would yield higher correlations with MSSC, while we would not expect any formalized measure to surpass subjective ratings of conceptual complexity.

Relatedly, MSSC does not address the gap in knowledge about complexity of naturalistic images of the kind we focused on in this work and complexity of abstract patterns (Bertamini et al., 2018; Chipman, 1977; Fitousi & Algom, 2024). For abstract patterns, apparent structure of many kinds has been shown to reduce complexity, as compared to unstructured patterns (Chipman, 1977). It has also been shown that reliance on structure in complexity judgments develops quite late, becoming fully apparent only at about 12 years of age (Chipman & Mendelson, 1979). A lot of progress has been made in quantifying complexity that emerges from structure in abstract patterns (Fitousi & Algom, 2024), yet it remains unclear how this knowledge can be applied to naturalistic images. An interesting attempt has been made by Taylor et al. (2008) who quantified “fractalness” (self-similarity) in naturalistic images and works of art (Pollock). Whether this approach is generalizable to any naturalistic image remains to be discovered.

Another promising avenue for further investigation is to take a more performance-centered approach to complexity of processing in humans, for example by estimating the time it takes to process an image, as well as the impact of task on perceived complexity (Cardaci et al., 2009; Jolicoeur et al., 1986).

### Future Directions

#### Informational and Effective Complexity

As reviewed in the introduction, complexity affects esthetic judgments and information exploration in a nonlinear manner (Berlyne, 1971; Oudeyer, 2018; Poli et al., 2024). It could be, however, that the nonlinearity is driven by the fact that complexity is usually measured as the amount of information, while humans intuitively define it as ‘‘effective complexity” (Gell-Mann & Lloyd, 1996), Figure 11. The former tends to be maximized at complete chaos/randomness, while the latter is maximized in the state of balance between order and randomness. With this distinction in mind, it seems sensible to revisit existing studies, testing whether the U-curve rule still holds when an effective complexity measure is applied instead of an informational one. Now that we have shown its correlation to human ranking, MSSC provides an opportunity for that.

**Figure 11.**
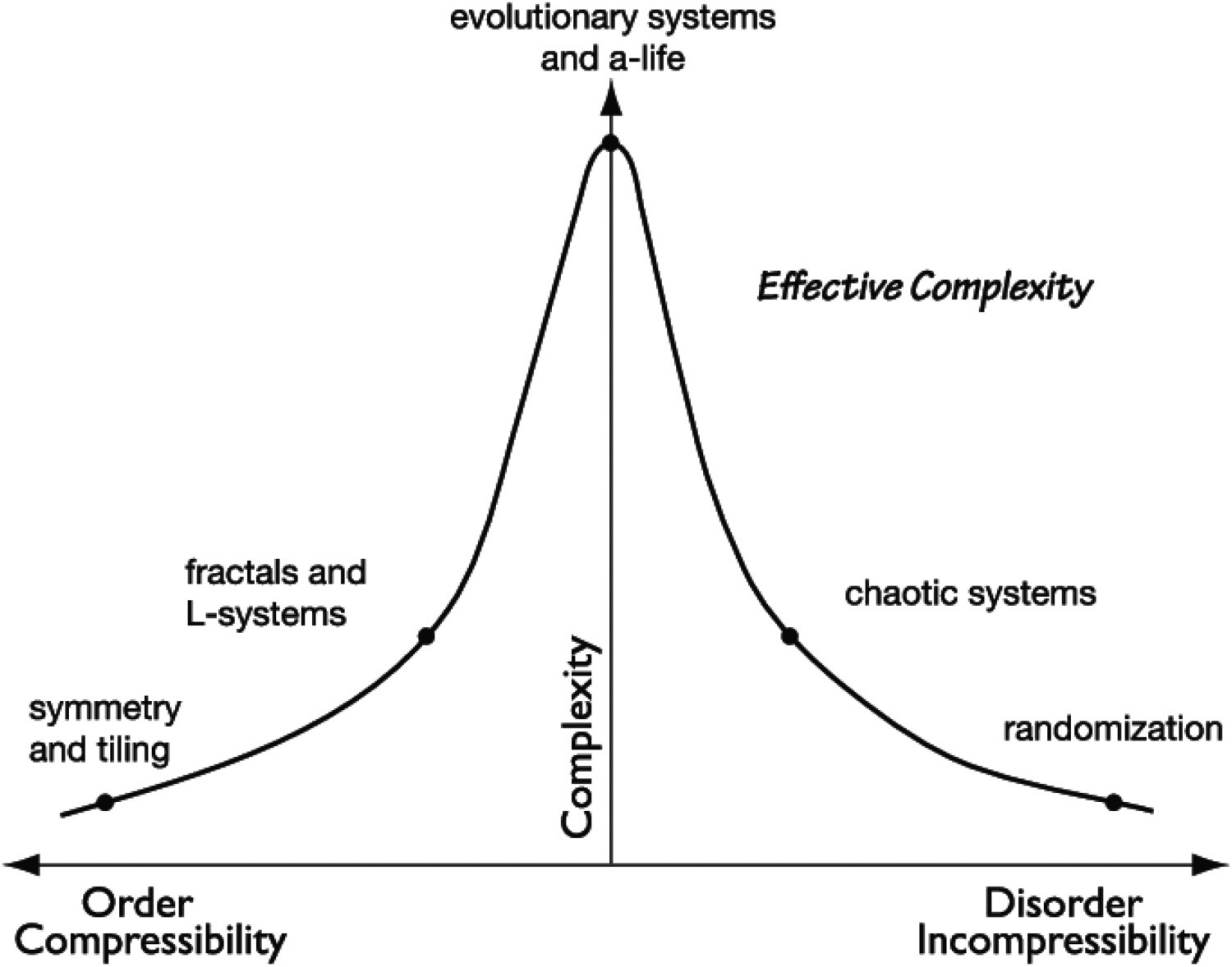
Informational and Effective Complexity. Image taken from Galanter (2019).

#### Partial Complexity

By breaking down self-dissimilarity at different scales of the image, MSSC opens the opportunity to specify different types of complexity via, for example, giving scales unequal weight in their contribution to overall complexity. More work is needed to test if complexity of different types of images (art vs. photographs of natural scenes) are best approximated by differently weighted MSSC.

#### Physiological Plausibility

MSSC is the first complexity measure that can implement different methods of information processing at the lowest level, which would correspond to the level of the retina, LGN, and V1 in human vision. In the present study, we used Fourier transform as a consensus approximation. Future studies could test other methods, such as wavelet analysis, and test the impact of different approaches to color processing.

#### Practical Applications

MSSC holds practical utility concerning design and infographics, particularly in studies exploring visual strain versus informational value within design contexts.

It has been shown that humans have an esthetic preference for natural scenes (Kaplan et al., 1972) and that low-level visual features and spatial properties have a significant effect on esthetic perception of scenes (Kardan et al., 2015). Considering the difference in the impact of spatial scales for man-made and natural images, studying the distribution of partial complexity could lead to clear guidelines or even automated testing of design and infographics.

## Conclusions

To summarize, compared to alternative approaches, MSSC offers advantages in terms of consistency of complexity metric across categories of images, computational feasibility, and physiological plausibility. It holds the potential to inform future research in curiosity, attention, and esthetic perception. Most importantly, it opens the venue for generating testable hypotheses about other, nonunitary conceptualizations of complexity.

## Appendix

### Coarse Graining-Procedure and Multiscale Complexity

To illustrate the coarse-graining procedure, let us focus on the case of grayscale images. Such an image can be viewed as an intensity function f(x) defined on some domain D (usually rectangular canvas), with f=1 corresponding to white color, and f=−1 corresponding to black color. The image can be coarse grained by convolving f(x) with some scale-dependent filter $g_{d\lambda }(x)$ to remove the features and details of size smaller than dλ :

(3)
$$f_{d\lambda }(x)=\int_{D}f(x)g_{d\lambda }(x).$$

If $f_{d\lambda }(x)$ is nearly identical to f(x) , it means that by removing small features from the pattern, we did not lose essential information, and there are no structures associated with scale dλ . In this case, we claim that this scale does not contribute to structural complexity. On the other hand, if the difference between f(x) and $f_{d\lambda }(x)$ is substantial, it is a situation similar to observed in real complex systems, when two different scales have rather distinct properties, and contribution of scale dλ to structural complexity can be regarded as large. To quantify this dissimilarity in the MSSC approach, nonnormalized overlap is introduced:

(4)
$$\langle f(x)|f_{d\lambda }(x)\rangle \equiv \int_{D}f(x)\cdot f_{d\lambda }(x).$$

Only the original pattern is normalized, so that ⟨f(x)|f(x)⟩=1 . Normalizing subsequent scales does not make much sense because the overall change in intensity over the course of coarse graining is also a part of overall dissimilarity between different scales, and must not be ignored.

The convolution procedure is subsequently applied a number of times with the same smearing parameter dλ , and the partial complexity associated with scale kdλ is defined as follows:

(5)
$$C_{k}=|\langle f_{kd\lambda }(x)|f_{(k+1)d\lambda }(x)\rangle$$

(6)
$$-\frac{1}{2}\left(\langle f_{kd\lambda }(x)|f_{kd\lambda }(x)\right\rangle$$

$$\begin{array}{rl} & +\left\langle f_{(k+1)d\lambda }(x)|f_{(k+1)d\lambda }(x)\rangle \right)|\\ & =\frac{1}{2}\int_{D}{\left(\;f_{(k+1)d\lambda }(x)-f_{kd\lambda }(x)\right)}^{2}dx. \end{array}$$

Summing up this over all scales, one obtain a number called *multiscale structural complexity* C :

(7)
$$C=\sum_{k}C_{k}.$$

Both C as an integral characteristic accounting for features emerging at every new scale, and the set of partial complexities $\left\{C_{k}\right\}$ contain important information about the pattern, and are usually used together.

In the case of color patterns, partial and overall complexities in each RGB channel are computed independently and then averaged. Each channel is scaled to [−1,1] range, with −1 representing the complete absence of the corresponding color, and +1 representing its maximal contribution.

### Implementation

The code for computing MSSC for images was developed from a code published by Bagrov et al. (2020), which was adapted and optimized for processing images. Most importantly, the present code uses Fourier transform for coarse graining, while Bagrov et al. used decimation based on averaging. Our motivation for this choice is explained in the main text, and we recognize that more work is needed to test alternative ways to estimate structural complexity in visual stimuli.
